# Immune Characterization of Ovarian Cancer Reveals New Cell Subtypes With Different Prognoses, Immune Risks, and Molecular Mechanisms

**DOI:** 10.3389/fcell.2020.614139

**Published:** 2020-12-21

**Authors:** Shanshan Cong, Qiuyan Guo, Yan Cheng, Yanan He, Xibo Zhao, Congcong Kong, Shangwei Ning, Guangmei Zhang

**Affiliations:** ^1^Department of Gynecology, The First Affiliated Hospital, Harbin Medical University, Harbin, China; ^2^College of Bioinformatics Science and Technology, Harbin Medical University, Harbin, China

**Keywords:** ovarian cancer, immune, tumor microenvironment, CIBERSORT, prognosis

## Abstract

Ovarian cancer (OV) is a considerable threat to the health of women due to its complex mechanisms and atypical symptoms. Various currently available treatments fail to substantially increase the survival rate of OV patients. The tumor microenvironment (TME) is gaining attention due to its role in tumorigenesis and tumor progression. This study mainly investigated the immune characteristics of OV by CIBERSORT and MCP-counter. We reclassified OV into four TME cell subtypes with different prognoses and evaluated the infiltration of the cells in each subtype. The immune risk of diverse subtypes was evaluated based on the immunoscore calculated by Cox regression analysis. The molecular mechanisms and hallmark pathways of the four subtypes were analyzed. The results indicate that the immune procancer cell subtype is associated with the worst prognosis, closely related to the high immune risk group, and characterized by low expression of checkpoints and MHC class I and II molecules, high expression of hypoxia-related genes, high enrichment of the EMT and hypoxia pathways, and low enrichment of the DNA repair and interferon α response pathways. This study contributes to the investigation of immune mechanisms and identifies more effective targets for immunotherapy of OV.

## Introduction

Ovarian cancer is a highly lethal malignancy that ranks as the seventh leading cause of female cancer worldwide; there will be approximately 21,750 new cases of OV in 2020 (National Cancer Institute, 2020). Therefore, OV is a considerable threat to the health and lives of women. Current treatments for OV mainly involve a combination of surgery and chemotherapy based on platinum and paclitaxel. Studies of OV have validated a number of new therapies (targeted therapy and immunotherapy) (Lamichhane et al., 2017; Reislander et al., 2019). However, due to drug resistance and recurrence of OV, the mortality of OV remains higher than that of all gynecological malignancies, and the 5-year survival rate of OV has kept at approximately 45% (Ghisoni et al., 2019). Therefore, new perspectives and in-depth investigation of OV are needed.

The tumor microenvironment (TME) is the complex environment of a tumor that is mainly composed of the vasculature, cells, and extracellular matrix (Henke et al., 2019; Baghban et al., 2020). Fibroblasts, endothelial cells, and immune cells (lymphocytes, monocytes, macrophages, granulocytes, mast cells, etc.) are the major cellular components of the TME (Quail and Joyce, 2013; Guo and Deng, 2018; Peltanova et al., 2019); these cells are known as tumor microenvironment cells (TME cells). TME cells closely interact with the tumor cells that consequently influence the local immune response and progression of the tumors. Studies on TME in OV have identified multiple TME cells that are relevant to the prognosis of OV (Drakes and Stiff, 2018). However, the majority of publications on TME cells in OV are based on only a single or a few cell types and rarely integrate all TME cells to define their relationship with OV.

Therefore, we aimed to analyze the immune characteristics and roles of TME cells in OV in detail. An important step in our study was to link the RNA-seq data to TME cells by CIBERSORT (Drakes and Stiff, 2018) and MCP-counter (Becht et al., 2016). CIBERSORT is the most frequently cited immune cell infiltration analysis tool based on the principle of deconvolution linking the expression of 547 genes to 22 types of immune cells. MCP-counter includes 10 cell types, and fibroblasts and endothelial cells were used in our study because all other cell types in MCP-counter overlap with the cell types of CIBERSORT. Thus, our study used these two algorithms to estimate the proportion of TME cells to reclassify OV into four new subtypes; then, the TME cell infiltration pattern and immune risk of each subtype were assessed, and relevant molecular mechanisms were investigated.

## Materials and Methods

### Acquisition of Gene Profiles and Clinical Datasets of OV

The Cancer Genome Atlas (TCGA) RNA-seq data and clinical characteristics of OV were obtained from UCSC Xena^1^. The format of the TCGA RNA-seq data was fragments per kilobase million (FPKM). The primary solid tumor samples with the detailed FIGO stage, histological grade, and overall survival (OS) were selected. To validate the results, two additional raw datasets GSE26193 and GSE9891 were downloaded from the Gene Expression Omnibus (GEO)^2^.

### OV Data Processing

The Ensembl IDs of the TCGA data were converted to gene names by the annotation dataset downloaded from the Ensembl genome browser^3^, and the probe IDs in the GEO data were processed using the relevant platform dataset. If multiple Ensembl or probe IDs matched a gene name, the average of all IDs was used to calculate the expression value of a gene. The FPKM OV data of TCGA were transformed to the transcripts per million (TPM) data format because TPM can be used to transform the RNA-seq expression data similar to the gene expression data (Vera Alvarez et al., 2019). The GEO OV data were normalized using the mas5 algorithm in the Affy R package (Gautier et al., 2004). All data used in the study were in a non-log format.

### Evaluation of the Proportion of TME Cells in OV

CIBERSORT and MCP-counter were used to transform the RNA-seq data into the proportion of TME cells. The TCGA data were reformatted as required for CIBERSORT^4^. LM22 was selected as the signature file, and 1,000 permutations were used to acquire 22 immune cell fractions in 359 OV samples; the sum of the 22 immune cell fractions of each sample was assumed to be 1. The *p*-values of all TCGA samples were calculated, and *P* < 0.05 indicated the predominance of the immune cells in the sample. Screening of the results based on *P* < 0.05 yielded 172 TCGA samples with a certain fraction of 21 immune cell types (one cell type was removed because the fraction was zero in the results). The MCP-counter R package was used to evaluate the expression of two additional TME cell types, fibroblasts and endothelial cells. Then, the z-score was used to normalize the data acquired by two separate methods, and the results were combined to finally obtain the proportion of 23 TME cell types in 172 OV samples.

### Clustering Methods

To determine a better classification method for investigating OV, consensus clustering of 172 OV data samples was performed by the ConsensusClusterPlus R package (Wilkerson and Hayes, 2010). The optimal inflection point (*k* = 4) of the elbow plot was used to define the best cluster number of OV samples. Finally, four new OV subtypes were defined based on TME cells; these subtypes were different from the stage or grade. Additionally, 23 TME cell types were also clustered into four groups by hierarchical clustering based on Euclidean distance and Ward’s linkage.

### Cox Regression Analysis of TME Cells

Univariate Cox regression analysis of the proportion of 23 TME cell types and OS was performed for each sample. Six TME cell types were selected according to the hazard ratio and *p*-value of the Cox regression results to generate an immune risk model based on linear regression (Hijazi et al., 2016).

$$immunoscore=\sum_{i=1}^{n}\beta_{j}Exp\left(cell_{i}\right)$$

In the model, *Exp(cell_*i*_)* corresponds to the proportion of TME cell*_*i*_* and β_*j*_ corresponds to the regression coefficient of cell*_*i*_* obtained by the Cox regression analysis. Each sample was assigned an immunoscore based on the model, and the median of all immunoscores was defined as a cutoff value to divide the OV data into the high and low immune risk groups.

### Survival Analysis

Kaplan-Meier analysis was used to determine the relationship between the four subtypes and OS and to evaluate the prognostic significance of various subtypes by the survival R package (Lin and Zelterman, 2002). The survival analysis of the two immune risk groups was performed to determine the OS differences between the groups.

### Gene Set Variation Analysis

Gene Set Variation Analysis (GSVA) was performed by the GSVA R package (Hanzelmann et al., 2013), which was used to calculate the GSVA score of each hallmark pathway in 172 TCGA OV samples. Each hallmark pathway was characterized by exclusive gene sets downloaded from GSEA^5^. GSVA converted the expression of the gene sets into the GSVA score of the corresponding hallmark pathway in each sample. A high GSVA score corresponds to the high enrichment of a hallmark pathway in a sample.

### Statistical Analysis

Two groups were compared using Student’s *t*-test, and multivariate groups were compared by Kruskal-Wallis test. The receiver operating characteristic curve (ROC curve) was used to evaluate the sensitivity and specificity of the immunoscore-based prediction of OS. The area under the curve (AUC) was calculated by the timeROC R package (Blanche et al., 2013) to acquire the best predicted indicator. All analyses were implemented in R 3.6, and all *p*-values in the analyses were considered significant at *P* < 0.05.

## Results

### The Immune Landscape of OV Identified Four New Cell Subtypes

A total of 359 primary solid tumor samples of OV with detailed clinical information were downloaded from the TCGA dataset (Table 1). The gene expression data of these samples were converted to the ratio of 22 immune cell types by CIBERSORT filtered by the *p*-value to obtain the results (*P* < 0.05 corresponding to a high proportion of immune cells compared to non-immune cells in the tumor tissue; Newman et al., 2015). Two additional cell types (fibroblasts and endothelial cells) were acquired using the MCP-counter R package. As a result, the final data included the proportion of 23 TME cell types in 172 OV samples (CD4^+^ naïve T cells were removed because the proportion of this cell type was zero). Detailed clinical characteristics and cell proportions are listed in Supplementary Table S1.

**TABLE 1:** The clinical features of 172 TCGA OV samples.

Clinical feature	Variable	n
Age	<58	78
	≥58	94
Stage	I	1
	II	12
	III	137
	IV	22
Grade	1	1
	2	17
	3	153
	X	1
Event	Alive	82
	Dead	90
Lymph node metastasis	Yes	59
	No	18
	Not available	95
Residual tumor diameter	No macroscopic	30
	1–10 mm	77
	11–20 mm	13
	>20 mm	29
	Not available	23

*The total number of each clinical feature is 172.*

**FIGURE 1:**
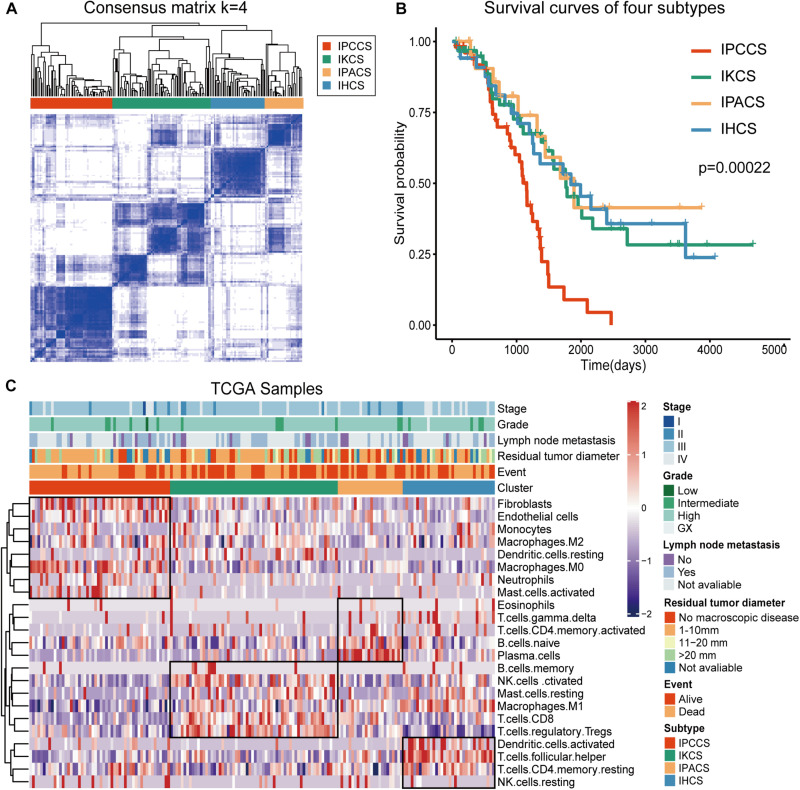
Division and analysis of four new cell subtypes in OV. **(A)** A clustergram of OV by the ConsensuSubtypePlus R package (*k* = 4). **(B)** The survival curves of four subtypes of OV. **(C)** The heatmap of 172 TCGA OV samples with four subtypes and clinical features.

To analyze the immune landscape of OV, consistent clustering was used to divide OV samples on the basis of the proportion of the 23 TME cell types. As a result, four new cell subtypes of OV were identified and named according to the functions of the cells: the immune procancer cell subtype (IPCCS, *n* = 52) (Kalluri and Zeisberg, 2006; Yuan et al., 2017; Lee et al., 2019), immune killer cell subtype (IKCS, *n* = 62) (Uzhachenko and Shanker, 2019), immune proantibody cell subtype (IPACS, *n* = 24) (Oracki et al., 2010), and immune helper cell subtype (IHCS, *n* = 34) (Banchereau and Steinman, 1998; Crotty, 2019; Figure 1A and Supplementary Table S2). The OS analysis indicated that IPCCS was associated with the poorest prognosis, and the other three subtypes were associated with similar or better prognosis than IPCCS (*P* < 0.001, Figure 1B). The landscape of TME cell infiltration in OV was used to assess the proportion of each cell type and detailed clinical features of 172 TCGA OV samples (Figure 1C). Therefore, IPCCS may be a new OV type that can predict prognosis regardless of clinical characteristics. TME cell types included in IPCCS were different from those in three other subtypes (Figure 1C). Thus, additional investigation of IPCCS is needed to identify the mechanism of association with poor OS.

### Different Cell Subtypes Manifest Specific TME Cell Infiltration Patterns

**FIGURE 2:**
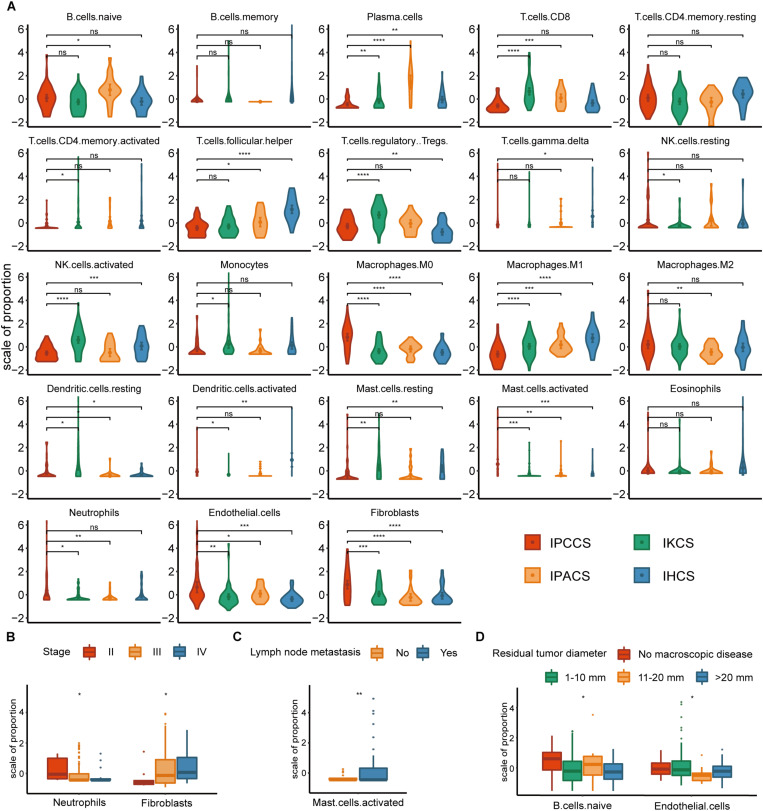
The distribution of TME cells in four subtypes and different clinical features of OV. **(A)** The proportion of each TME cell type in four subtypes of OV. **(B)** The distribution of neutrophils and fibroblasts in stages II, III, and IV of OV. **(C)** The distribution of activated mast cells in the lymph node metastases of OV. **(D)** The distribution of naïve B cells and endothelial cells in various residual tumor diameter of OV (statistical comparison between the two groups was performed by Student’s *t*-test, and multivariate groups were compared by Kruskal-Wallis test. ns *P* > 0.05, *P < 0.05, **P < 0.01, ***P < 0.001, ****P < 0.0001).

Significant discrepancies in IPCCS and the other three subtypes were identified in the case of 20 out of 23 TME cell types (Figure 2A and Supplementary Figure S1). M0 macrophages, M2 macrophages, activated mast cells, neutrophils, endothelial cells, and fibroblasts were considerably enriched in IPCCS, but a relatively low proportion of M1 macrophages and CD8^+^ T cells was found. IKCS was characterized by a high proportion of activated NK cells, CD8^+^ T cells, and regulatory T cells (Tregs). IPACS included a high number of naïve B cells and plasma cells. Activated dendritic cells, follicular helper T cells, and resting memory CD4^+^ T cells demonstrated high infiltration in IHCS, and Tregs were characterized by low infiltration.

Not all *p*-values corresponding to M2 macrophages were significant; however, the expression of M2 macrophages was high in IPCCS similar to that of M0 macrophages (Figure 2A). The proportion of M2 and M1 macrophages demonstrated an opposite trend. This phenomenon confirmed that macrophage polarization to M1 and M2 may have different effects on the occurrence and development of cancer (Cheng et al., 2019). CD8^+^ T cells and NK cells destroy cancer cells, play a critical role in immune effector activity and are advantageous to the outcome of multiple tumors (McGrail et al., 2018). These observations are similar to our findings that CD8^+^ T cells and activated NK cells were low expression in IPCCS, which is associated with the worst prognosis of all four subtypes. Thus, CD8^+^ T cells and activated NK cells may kill tumor cells in OV, and a new cell subtype identified by us can predict the prognosis of OV based on the proportion of TME cell types. Cancer-associated fibroblasts (CAFs) are important TME cells that release factors and boost angiogenesis leading to poor tumor outcome and resistance to treatment (Gascard and Tlsty, 2016). Our data indicated that CAFs were considerably increased in IPCCS, which was associated with the worst prognosis.

The distribution of TME cell types in relation to various OV clinical features (FIGO stage, histological grade, lymph node metastasis, and residual tumor diameter) was investigated. The results indicated that neutrophils and fibroblasts were distributed in stages II, III, and IV of OV (Figure 2B). These two cell types have completely opposite trends. Lee et al. (2019) reported that an increase in neutrophils in the premetastatic omental niche could be stimulated in early stage of OV. Zhao et al. (2017) showed that fibroblasts are characterized by high expression of CXCL 14 that is overexpressed in advanced-stage OV. Activated mast cells were present in variable proportions in lymph node metastasis of OV (Figure 2C). Crivellato et al. (2008) showed that mast cells can stimulate tumor angiogenesis and lymphangiogenesis and that the inhibition of mast cells decreased the lymph node metastasis. At the same time, naïve B cells and endothelial cells had different distribution in various residual tumor diameters (diameter of residual tumor in the pelvic and abdominal cavity after surgical treatment of OV) (Figure 2D). Cecilia S. Leung et al. demonstrated that endothelial cells can promote tumor progression and chemoresistance (Leung et al., 2018) to subsequently increase the probability of residual tumor.

### Certain Distinctive TME Cells Contribute to Diverse Immune Risk Grouping of Cell Subtypes

**FIGURE 3:**
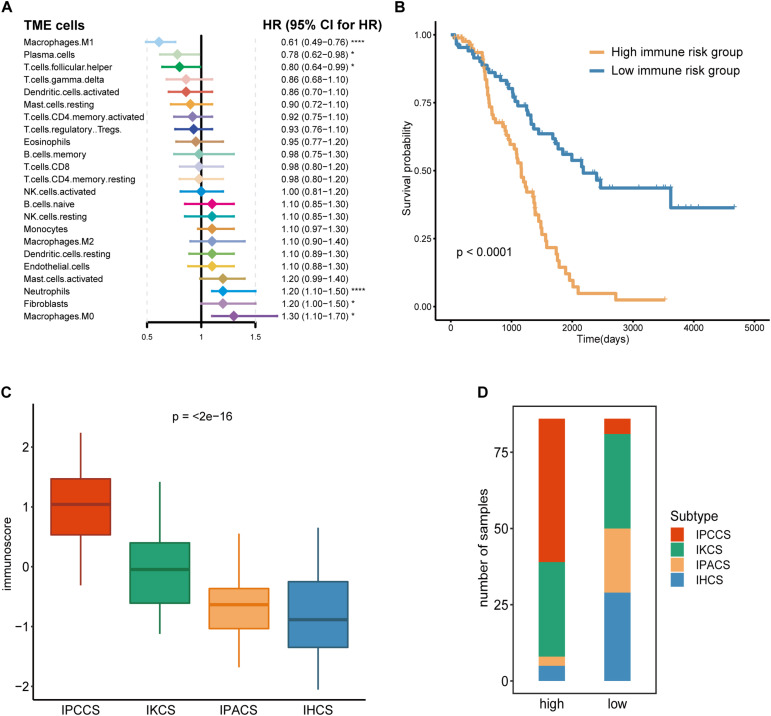
Calculation and application of the immunoscore. **(A)** The Cox regression analysis of 23 TME cell types of OV. **(B)** The survival curves of the immunoscore in OV. **(C)** The distribution of the immunoscore in four subtypes of OV. **(D)** The number of four subtypes of OV samples in the high and low immunoscore groups (statistical comparison between the multivariate groups was performed by Kruskal-Wallis test. *P < 0.05, ****P < 0.0001).

Given that TME cells are closely associated with the tumor, we generated a model that used TME cells to predict the immune risk of OV. Cox regression analysis identified six TME cell types (M1 macrophages, plasma cells, follicular helper T cells, neutrophils, fibroblasts, and M0 macrophages) with *P* < 0.05 according to the results of the analysis. These six TME cell types were used to establish an immune risk model of OV, and the results are shown as a forest plot (Figure 3A). The risk model included the results of the fraction levels multiplied by the regression coefficient of each of the six cell types. The immunoscore of 172 OV samples was calculated, and the median was used to separate the samples into the high and low immune risk groups (Supplementary Table S3). The Kaplan-Meier survival analysis of these two groups demonstrated considerable differences, and the high immune risk group was associated with poor OS (Figure 3B).

Comparison of the immunoscores of four subtypes indicated that IPCCS had the highest immunoscore and that the immunoscore of the four subtypes was gradually and slightly decreased (Figure 3C). This result is in agreement with the data of our previous survival analysis. IPCCS had the highest proportion in the high immune risk group and the lowest percentage in the low immune risk group, which was opposite to that of IPACS and IHCS (Figure 3D). The results indicated that IPCCS had a positive correlation with the high immune risk group. In contrast, IPACS and IHCS were positively correlated with the low immune risk group.

### Different Subtypes Have Characteristics of Various Molecular Mechanisms and Pathways

**FIGURE 4:**
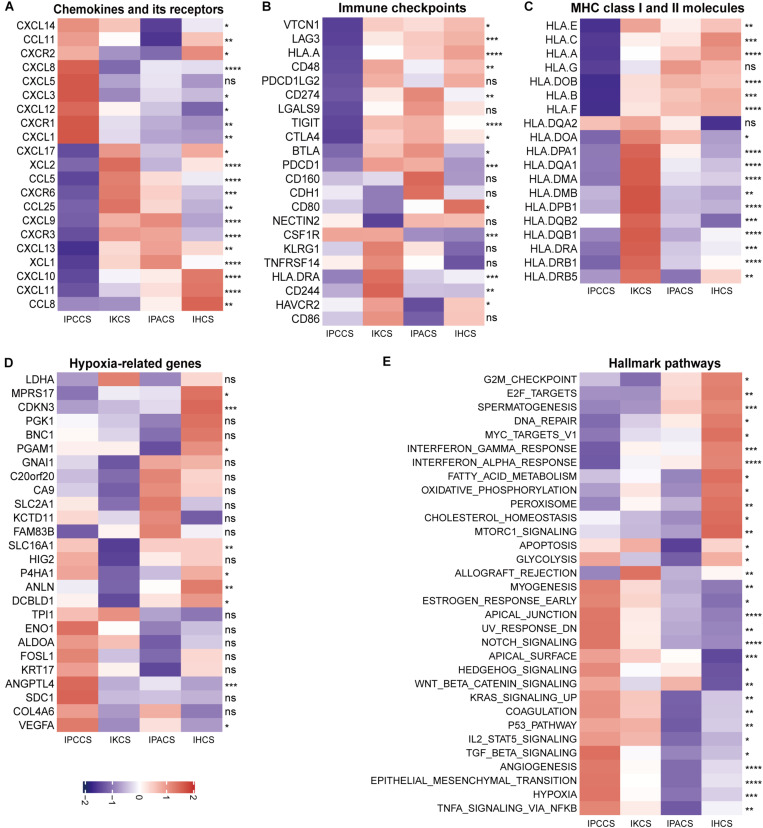
The expression of various molecules and hallmark pathways in the four subtypes. **(A)** The expression of chemokines and chemokine receptors in the four subtypes. **(B)** The expression of immune checkpoints in the four subtypes. **(C)** The expression of MHC class I and II molecules in the four subtypes. **(D)** The expression of hypoxia-related genes in the four subtypes. **(E)** The GSVA score of the hallmark pathways in the four subtypes (statistical comparison between the multivariate groups was performed by Kruskal-Wallis test. ns *P* > 0.05, *P < 0.05, **P < 0.01, ***P < 0.001, ****P < 0.0001).

To analyze the molecular mechanisms corresponding to various subtypes, current literatures were searched for data on chemokines and chemokine receptors (Nagarsheth et al., 2017), immune checkpoints (Huang et al., 2017), MHC class I and II molecules (Johnson et al., 2018), and hypoxia-related genes (Eustace et al., 2013). Initially, the expression of chemokines and chemokine receptors was compared in the four subtypes and two immune risk groups. The results showed that CXCL8, CXCL5, CXCL3, CXCL12, CXCR1, and CXCL1 were expressed at high levels in IPCCS and the high immune risk group (Figure 4A and Supplementary Figure S2A). Certain studies demonstrated that these chemokines or chemokine receptors are associated with the aggregation and activation of macrophages (Qin et al., 2017; Roca et al., 2018), neutrophils (De Filippo et al., 2013), and fibroblasts (Feig et al., 2013; Naito et al., 2019), which were also enriched in IPCCS; these factors may in part account for different TME cell infiltration patterns.

The majority of immune checkpoints were expressed at low levels in IPCCS and the high immune risk group (Figure 4B and Supplementary Figure S2B), especially including well-known PD-1 (PDCD1), PD-L1 (CD274), LAG-3, and CTLA-4 molecules, similar to those described in a study of Xiao et al. (2019). Thus, checkpoints do not play an important role in the progression of OV in IPCCS. Hence, we hypothesized that the effect of immunotherapy with checkpoint blockers in IPCCS may not be as pronounced as that in the other subtypes. The expression of most MHC class I and II molecules was also low in IPCCS and the high immune risk group (Figure 4C and Supplementary Figure S2C). Low proportion of various T cells in IPCCS may suggest that T cells cannot proliferate and recognize tumor cells due to low expression of MHC class I and II molecules.

Most hypoxia-related genes were expressed at high levels in IPCCS and the high immune risk group indicated a certain degree of hypoxia in the environment of OV cells (Figure 4D and Supplementary Figure S2D). Bhandari et al. (2019) estimated that OV has a higher hypoxia score, and hypoxia contributes to deterioration and chemotherapy resistance of OV (Dorayappan et al., 2018). Therefore, hypoxia may influence the proportion of TME cells and lead to poor prognosis of OV.

To determine the differences in the enrichment of hallmark pathways between various subtypes and immune risk groups, GSVA of the hallmark pathways was performed using 172 TCGA OV data. The GSVA score of the hallmark pathways demonstrated that certain pathways (such as EMT, TNFα signaling via NF-kB, hypoxia, angiogenesis, and notch signaling) were highly enriched in IPCCS and the high immune risk group (Figure 4E and Supplementary Figure S2E). These well-known pathways are known to be related to cancer progression (Chi et al., 2006; Takebe et al., 2014; Ottevanger, 2017; Markowska et al., 2017; Yang et al., 2018). In contrast, some pathways (such as DNA repair, spermatogenesis, oxidative phosphorylation, interferon γ response, and interferon α response) had negative effects in IPCCS and the high immune risk group compared to those in other subtypes and the low immune risk group (Figure 4E and Supplementary Figure S2E). Differential enrichment of these pathways may be used as an indicator of the mechanism of differences in immune infiltration and to improve individualized treatment of the patients with various subtypes and immune risks.

### Verification of Immune Risk Grouping in Independent Cohorts

**FIGURE 5:**
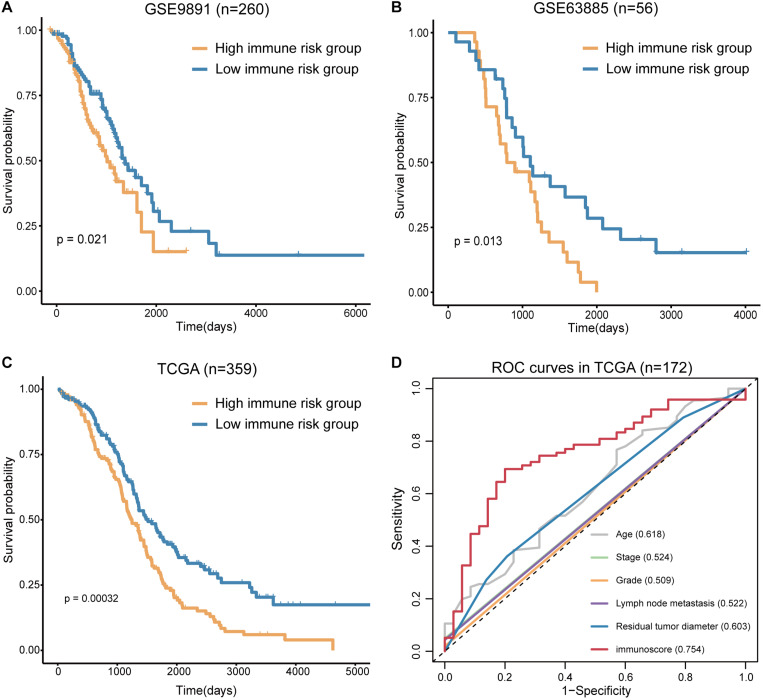
Verification of the immunoscore in independent cohorts. **(A)** The survival curves of immunoscore in GSE9891. **(B)** The survival curves of immunoscore in GSE63885. **(C)** The survival curves of immunoscore in TCGA OV. **(D)** The ROC curves of the clinical features and immunoscore in TCGA OV (numbers after the clinical features and immunoscore represent the corresponding AUC).

To confirm the prognostic accuracy of immune risk grouping, the model was applied in two independent GEO cohorts (GSE63885 and GSE9891) and the entire TCGA cohort (*n* = 359). The same six TME cell types were used to compute the immunoscore of each sample in the GEO and TCGA cohorts, and each cohort was divided into the high and low immune risk groups according to the median immunoscore (Supplementary Table S4). We found significant differences in the OS of the high and low immune risk groups in the GEO and TCGA cohorts (Figures 5A–C). The survival analysis indicated that the high immune risk group had worse prognosis similar to our previous results.

The sensitivity and specificity of the clinical features and immunoscore in predicting OS were evaluated in 172 TCGA samples. The results of the ROC curves analysis indicated that the AUC of the immunoscore was gradually increased from 1 to 5 years (Supplementary Figure S3), and the immunoscore had the largest AUC compared to those of all clinical features (Figure 5D). Thus, the immunoscore had the highest associations with predicted OS in the TCGA OV samples. In summary, the immune risk grouping developed in the present study can be a powerful means to evaluate the prognosis of OV.

## Discussion

Previous tumor studies have focused on aberrant genes, epigenetics, or non-coding RNA. Subsequent studies have gradually revealed associations between inflammation, immune cells, and the TME and tumorigenesis and tumor development. The effect of the TME on tumors is a complex and dynamic process. The proportion and activation status of TME cells differentially influence the proliferation, invasion, and metastasis of tumor cells (Quail and Joyce, 2013). Therefore, we investigated the effect of the TME on OV by a new approach designed in the present study.

Current studies on the TME of OV are based on a single or a few cell types; the expression of these cell types depends mainly on detecting several specific gene markers. Our study defined 21 TME cell types by CIBERSORT and 2 TME cell types by MCP-counter thus expanding the data coverage of our study of the TME of OV. Each cell type had dozens of representative genes that can increase the accuracy of the estimation of their expression. MCP-counter was selected mainly because it includes fibroblasts and endothelial cells, which have been shown to play key roles in the occurrence and development of cancer (Maishi and Hida, 2017; Thuwajit et al., 2018).

CIBERSORT and MCP-counter analyses of the TCGA OV data yielded the proportions of 23 TME cell types of OV. A classification diagram was used to divide all OV data into four subtypes based on the proportion of 23 TME cell types indicating that TME of OV has four main categories. These subtypes were different from the routine stage and grade characteristics, and each subtype had unique cell types that may account for different prognosis of OV. The Cox regression analysis of these cell types was used to select six TME cell types (*P* < 0.05) to construct an immune risk model. The model was used to calculate the immunoscore and evaluate the immune risk in each OV sample. The results indicated that different cell subtypes of OV are associated with variable immune risk. IPCCS was associated with high immune risk and the worst prognosis. In contrast, IPACS and IHCS were associated with low immune risk and better prognosis. The data indicated that the model can reliably distinguish differences in the survival time of the patients in the high and low immune risk groups; the high immune risk group was associated with poor prognosis. Consistent results were obtained by validating the model using the GEO database. The ROC curve analysis also showed that the immunoscore had higher sensitivity and specificity in the prediction of prognosis compared with that of other clinical features.

Previous studies demonstrated that chemokines and chemokine receptors play positive roles in inflammation and oncogenesis by regulating the trafficking of various inflammatory cells (Bian et al., 2019). Immune checkpoints play an important role in TME and can be potential targets for cancer treatment (Toor et al., 2020). MHC I and II molecules can present protein fragments to T cells that are essential for cell-mediated immunity and tumor immunity (Rock et al., 2016; Alspach et al., 2019). At the same time, hypoxia is frequent in TME and can change the components of TME leading to poor prognosis of cancer (Jing et al., 2019). Therefore, these genes are related to TME and have to be considered in the studies of immune characteristics of OV. The present study evaluated the expression of the chemokines, chemokine receptors, immune checkpoints, MHC class I and II molecules, hypoxia-related genes, and enrichment of the hallmark pathways in four subtypes to determine the mechanisms of different immune infiltration patterns. The results indicated that differences in TME cell infiltration in the subtypes were closely related to the expression of the chemokines and chemokine receptors because of the characteristics of these molecules that induced directional migration of immune cells (Sokol and Luster, 2015). High expression of the hypoxia-related genes in IPCCS was caused by hypoxic conditions in the TME (Jing et al., 2019). Low expression of immune checkpoints (such as PD-1, LAG-3, CTLA-4) which mainly exist on the surface of T cells (Dyck and Mills, 2017) in IPCCS mainly due to the lower proportion of various T cells in this subtype than the other three subtypes (IKCS, IPACS, and IHCS). Studies stated that checkpoints blockade might as new targets for cancer immunotherapy (Feng et al., 2019). Therefore, we could speculate that the therapy of checkpoints blockade might have better effects in the other three subtypes of OV. Numerous studies demonstrated that a number of pathways are linked to the progression and prognosis of OV (Wang et al., 2017; Garsed et al., 2018; Guo et al., 2018; Zeng et al., 2018). We compared differential enrichment of the hallmark pathways in four subtypes, and the results indicated that the mechanisms of the action of different cell subtypes are related to different pathways.

Although the OV dataset used in the present study was downloaded from TCGA, the number of OV samples was cut by almost 50% by CIBERSORT. In the future, the collection of additional clinical data on OV, corresponding clinical characteristics, and survival time is needed to increase the accuracy of our study. Our findings may require verification in additional investigations of TME cells and pathways. Differences in gene mutations, expression of ncRNAs, copy number variations, and other factors in the four subtypes require further studies.

## Conclusion

In summary, our study reclassified OV into four subtypes (IPCCS, IKCS, IPACS, and IHCS) according to TME cell types and demonstrated infiltration of exclusive cell types in each subtype of OV. Then, six TME cell types were selected by Cox regression analysis to calculate the immunoscore that could assess the immune risk and predict the prognosis of OV. The results showed that IPCCS was associated with high immune risk and poor prognosis. Finally, the analysis of the mechanisms of various subtypes of OV was performed, and the results may assist in identifying effective therapeutic targets for OV.

## Data Availability Statement

Publicly available datasets were analyzed in this study. This data can be found here: http://xena.ucsc.edu; https://www.ncbi.nlm.nih.gov/geo.

## Author Contributions

GZ and SN designed and directed all research. SC, QG, YC, YH, XZ, and CK performed the data processing and experimental analyses. GZ, SN, SC, and QG drafted the manuscript. All authors reviewed and approved the final version of the manuscript.

## Conflict of Interest

The authors declare that the research was conducted in the absence of any commercial or financial relationships that could be construed as a potential conflict of interest.

## Footnotes

1 http://xena.ucsc.edu

2 https://www.ncbi.nlm.nih.gov/geo

3 https://uswest.ensembl.org

4 https://cibersort.stanford.edu

5 https://www.gsea-msigdb.org
