# Supplementary figures and images for: Immune Characterization of Ovarian Cancer Reveals New Cell Subtypes With Different Prognoses, Immune Risks, and Molecular Mechanisms
Source: Front Cell Dev Biol. 2020 Dec 21;8:614139. doi: 10.3389/fcell.2020.614139 (PMC7779527; doi:10.3389/fcell.2020.614139)

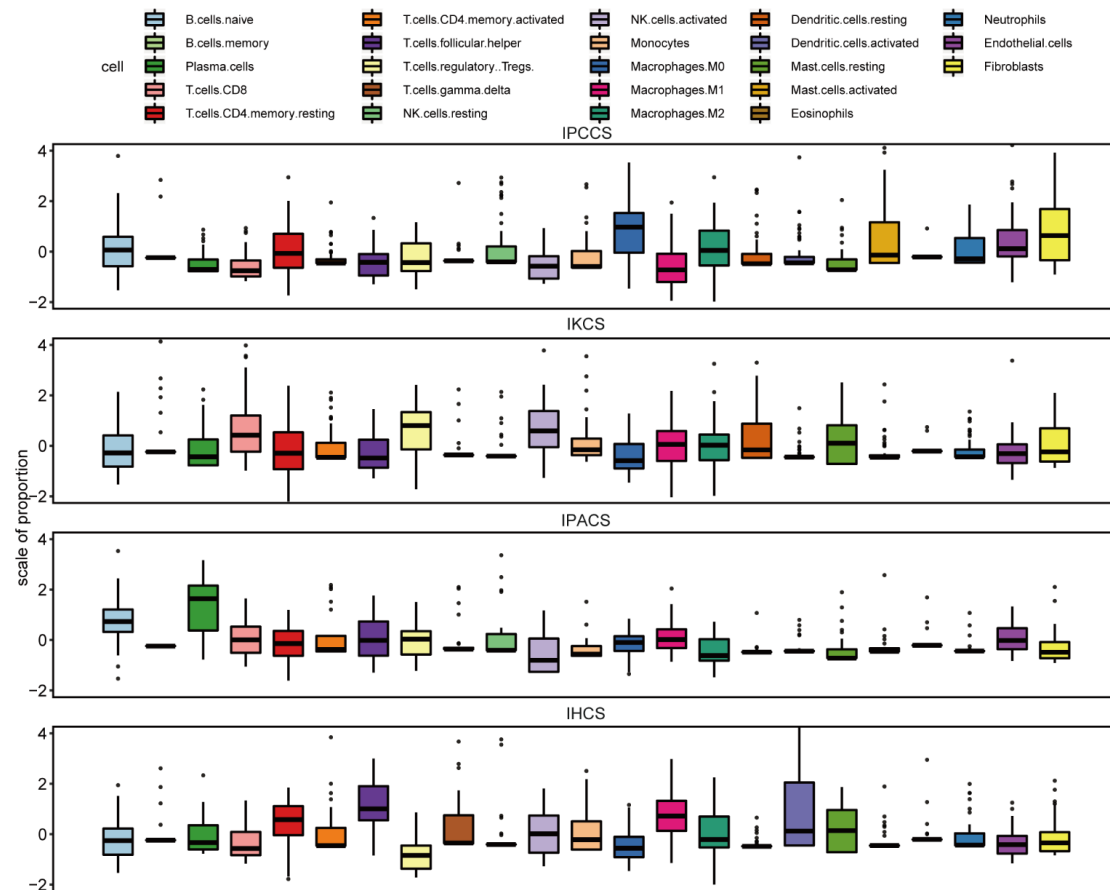

**Supplementary Figure 1.** The proportion of 23 TME cells in each subtype of OV.

Supplement: Supplementary file 5 [file Data_Sheet_1.PDF]
