# Supplementary material for: Immune Characterization of Ovarian Cancer Reveals New Cell Subtypes With Different Prognoses, Immune Risks, and Molecular Mechanisms
Source: Front Cell Dev Biol. 2020 Dec 21;8:614139. doi: 10.3389/fcell.2020.614139 (PMC7779527; doi:10.3389/fcell.2020.614139)
Supplement: Supplementary file 6 [file Data_Sheet_2.PDF]

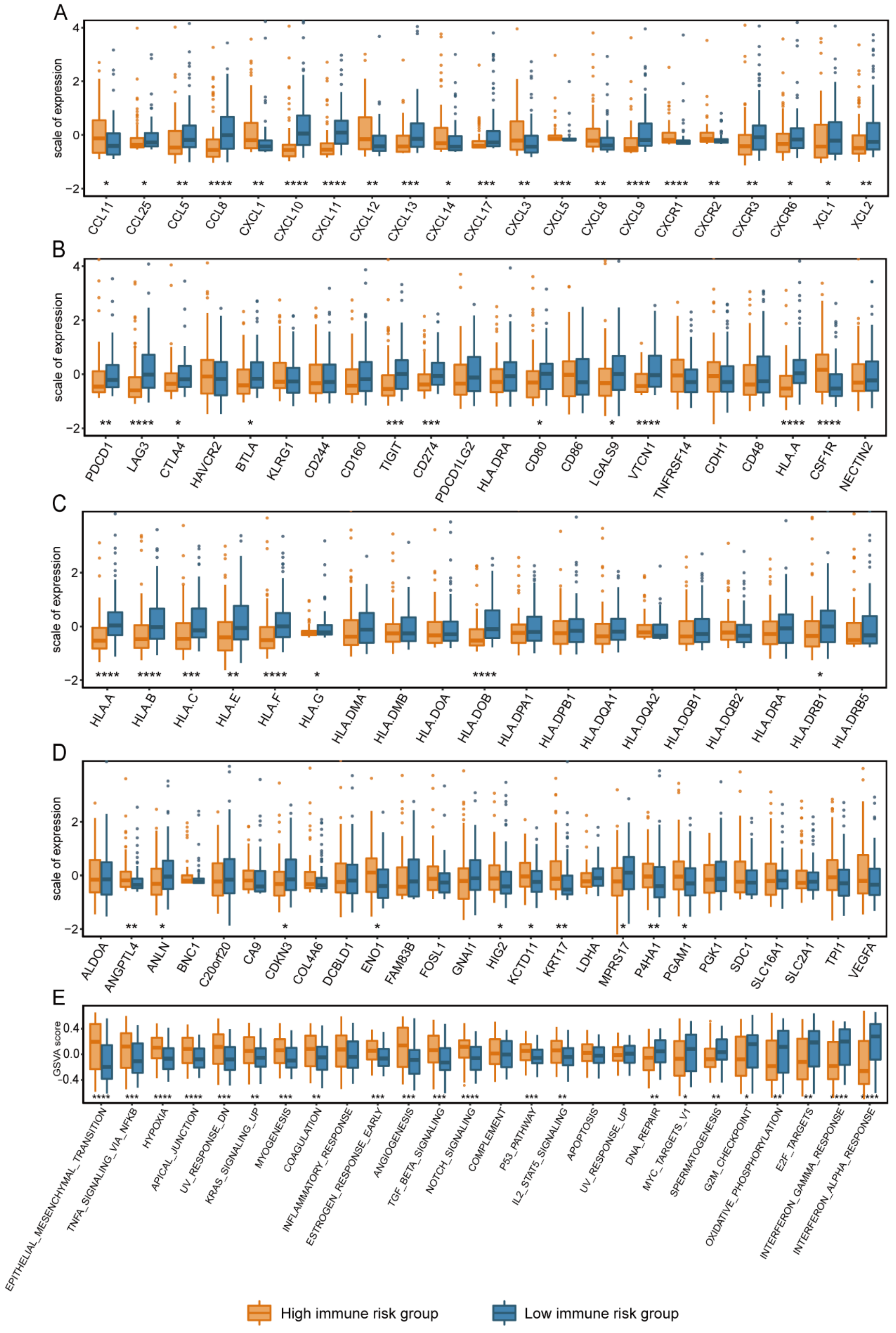

Supplementary Figure 2. The expression of various molecules and hallmark pathways in the high immune risk group and the low immune risk group. (**A**) The expression of

chemokines and chemokine receptors in the high immune risk group and the low immune risk group. (**B**) The expression of immune checkpoints in the high immune risk group and the low immune risk group. (**C**) The expression of MHC class I and II molecules in the high immune risk group and the low immune risk group. (**D**) The expression of hypoxia-related genes in the high immune risk group and the low immune risk group. (**E**) The GSVA score of hallmark pathways in the high immune risk group and the low immune risk group. (The statistical method between two groups was Student's *t* test. *$P<0.05$; **$P<0.01$; ***$P<0.001$; ****$P<0.0001$)
