# Supplementary material for: Immune Characterization of Ovarian Cancer Reveals New Cell Subtypes With Different Prognoses, Immune Risks, and Molecular Mechanisms
Source: Front Cell Dev Biol. 2020 Dec 21;8:614139. doi: 10.3389/fcell.2020.614139 (PMC7779527; doi:10.3389/fcell.2020.614139)
Supplement: Supplementary file 7 [file Data_Sheet_3.PDF]

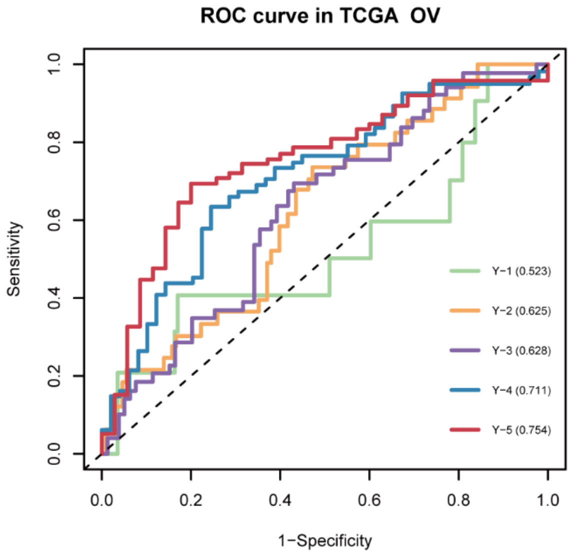

Supplementary Figure 3. The 1-5 years ROC curves of immunoscore in TCGA OV samples (The numbers following the years represent their corresponding AUC).
